# Living apart together: Long-term coexistence of Baltic cod stocks associated with depth-specific habitat use

**DOI:** 10.1371/journal.pone.0274476

**Published:** 2022-09-28

**Authors:** Franziska Maria Schade, Peggy Weist, Jan Dierking, Uwe Krumme

**Affiliations:** 1 Thünen Institute of Baltic Sea Fisheries, Rostock, Germany; 2 Thünen Institute of Fisheries Ecology, Bremerhaven, Germany; 3 GEOMAR Helmholtz Centre for Ocean Research, Kiel, Germany; Swedish University of Agricultural Science, SWEDEN

## Abstract

Coexistence of fish populations (= stocks) of the same species is a common phenomenon. In the Baltic Sea, two genetically divergent stocks of Atlantic cod (*Gadus morhua*), Western Baltic cod (WBC) and Eastern Baltic cod (EBC), coexist in the Arkona Sea. Although the relative proportions of WBC and EBC in this area are considered in the current stock assessments, the mixing dynamics and ecological mechanisms underlying coexistence are not well understood. In this study, a genetically validated otolith shape analysis was used to develop the most comprehensive time series of annual stock mixing data (1977–2019) for WBC and EBC. Spatio-temporal mixing analysis confirmed that the two stocks coexist in the Arkona Sea, albeit with fluctuating mixing proportions over the 43-year observation period. Depth-stratified analysis revealed a strong correlation between capture depth and stock mixing patterns, with high proportions of WBC in shallower waters (48–61% in <20m) and increasing proportions of EBC in deeper waters (50–86% in 40-70m). Consistent depth-specific mixing patterns indicate stable differences in depth distribution and habitat use of WBC and EBC that may thus underlie the long-term coexistence of the two stocks in the Arkona Sea. These differences were also reflected in significantly different proportions of WBC and EBC in fisheries applying passive gears in shallower waters (more WBC) and active gears in deeper waters (more EBC). This highlights the potential for fishing gear-specific exploitation of different stocks, and calls for stronger consideration of capture depth and gear type in stock assessments. This novel evidence provides the basis for improved approaches to research, monitoring and management of Baltic cod stocks.

## Introduction

The frequent coexistence of fish populations of the same species challenges our understanding of population structure and dynamics [1]. Closely related yet genetically different populations living in the same area often compete for limited resources, e.g. food, shelter or appropriate environmental conditions. Stable coexistence of populations is typically attributed to niche segregation, i.e. competing populations develop different life history strategies to coexist [2]. Some fish populations coexist in the same area only for a relatively short term, for instance, during seasonal feeding periods (e.g. herring: [3]), while other fish populations coexist during their entire lifespan and have specialised in different diets (e.g. cod: [4]) or colonized different depth zones (e.g. hake: [5]).

**Fig 1:**
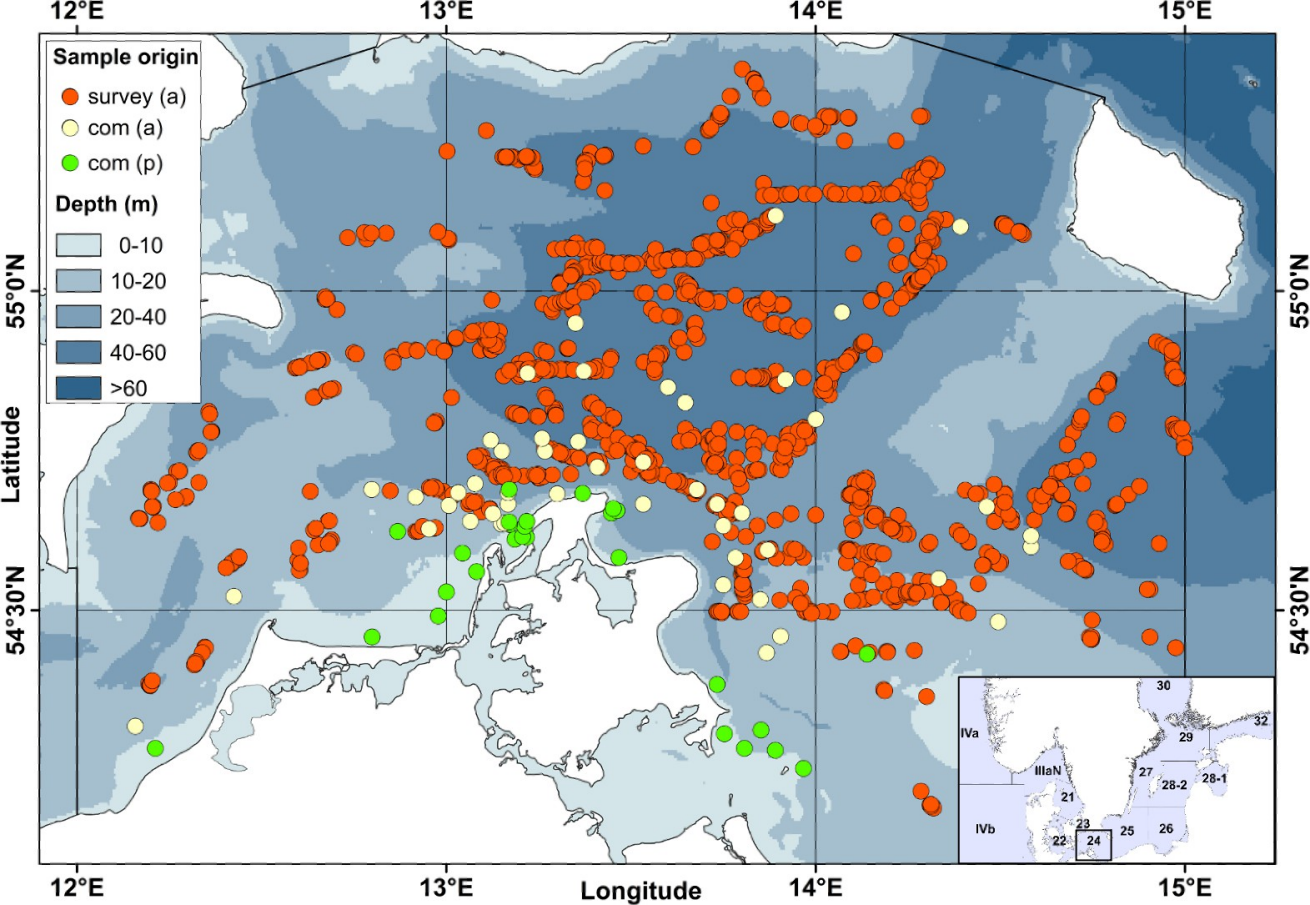
Sampling locations of Baltic cod in SD 24. Cod samples originate from bottom-trawl survey catches (1977 to 2019) and commercial (= com) catches (2010 to 2019) using active (= a) and passive (= p) fishing gears. Inset is a map of the larger Baltic Sea region, indicating ICES subdivisions 22 (Belt Sea), 23 (Øresund), 24 (Arkona Sea) and 25 (Bornholm Basin). Grid within SD 24 represents ICES statistical rectangles. Source of elevation data from [41].

The Atlantic cod (*Gadus morhua*, Gadidae) is a fish species of high ecological and economical importance in the North Atlantic, which inhabits also the brackish waters of the Baltic Sea. Two distinct cod populations (= stocks) exist in the Baltic Sea, i.e. the Western Baltic cod (WBC, ICES subdivisions (SDs) 22–24) and the Eastern Baltic cod (EBC, SDs 24–32, Fig 1; [6]). The stocks are genetically divergent [7, 8] and differ in main spawning times and grounds [9]. WBC spawn mainly in spring in the shallower western Baltic Sea (SDs 22 and 23), while EBC spawn mainly in summer in the deeper basins of the eastern Baltic Sea (SDs 25 and 26; [9, 10]).

The cod stocks also differ in several other parameters, e.g. the WBC stock has a lower stock size [11] but cod in the western Baltic Sea have a higher growth rate and reach larger sizes [12, 13], their livers are less infested with anisakid larvae [14] and they have more easily readable otoliths [15].

Historical Baltic cod tagging experiments [16, 17] and recent genetic data [7, 8] detected a substantial mixing of the cod stocks, particularly in the Arkona Sea (SD 24). The frequent occurrence of WBC and EBC in the same fishing hauls in collections for these studies indicate that the two stocks live in close association in SD 24. Although stock affiliation of Baltic cod is considered in current stock assessments and practical fisheries management in SD 24 [6, 18], the ecological mechanisms underlying coexistence and the long-term dynamics of stock mixing in this area are still unknown.

Coexistence of cod populations is a common phenomenon in the North Atlantic [1, 19, 20]. Cod populations in Iceland and Norway, for instance, coexist in the same area by displaying stationary and long-distance migratory behaviours [1, 21, 22]. Stationary cod frequent shallower waters and more coastal habitats, while migratory ecotypes exploit deeper waters and more offshore habitats [19].

To understand mixing dynamics and ecological drivers of the coexistence of populations, long-term mixing data at multiple spatial scales are needed. For stock mixing analysis, individuals from mixed-stock catches need to be assigned to their stock of origin using reliable stock discrimination methods with high assignment accuracy [23–25]. Genetic approaches are favoured to unequivocally assign individual fish with unknown origin to its respective stock [26, 27], but they are still time-consuming, costly and only applicable when tissue samples are available. Given the availability of extensive otolith archives in fisheries research institutes, otolith-based stock discrimination methods may allow to reconstruct mixing dynamics over longer time periods [28, 29].

Otolith shape analysis is a common stock discrimination method in fisheries science [25, 30, 31] and has been successfully used to discriminate Baltic cod stocks with a high assignment accuracy [23, 24, 29]. So far, only one study has investigated spatio-temporal patterns in stock mixing of Baltic cod in SD 24 by analysing the shape of otoliths from 1996 to 2014 [28]. However, the study covered only a short period and had gaps in the time series, i.e. only 9 out of 19 years were analysed. Moreover, the spatial coverage of mixing data was limited, e.g. there were just a few samples available for the area 13° to 14°E and no samples from the shallower southern area in SD 24. The determined mixing proportions of Baltic cod stocks may thus only be representative for the area studied but not for the entire SD 24.

In the 1980s, the spawning stock biomass (SSB) of both Baltic cod stocks was at a record high with >40 000 t for WBC and >400 000 t for EBC [6]. In subsequent years, the SSB of EBC strongly declined below 100 000 t due to high fishing pressure and unfavourable environmental conditions for recruitment [32]. Despite some periods of stock recovery [33], the EBC stock is currently in a critical state and under distress [34, 35]. The SSB of WBC also decreased significantly over the decades and reached a historic low in recent years [35]. Overlaying these long-term trends, both stocks have also displayed strong interannual fluctuations in SSB and catches. This raises the question of whether and how the major changes in the respective abundances of the two stocks in the western and eastern Baltic Sea have affected the mixing dynamics in the Arkona Sea over time.

In this study, our aim was to address the current knowledge gaps, by applying a combined approach of a genetically validated baseline and otolith shape analysis to develop a multi-decadal time series of annual stock mixing data of Baltic cod in SD 24. The specific objectives were to (i) investigate long-term spatio-temporal patterns of stock mixing in the Arkona Sea (SD 24), with a closer focus on (ii) the depth-specific patterns of stock mixing. To evaluate potential differences in the stock composition of catches from different fishing fleets, we also (iii) examined fisheries-specific stock mixing data from commercial catches using active and passive fishing gears. We discussed these results in the light of potential ecological mechanisms underlying coexistence of WBC and EBC, as well as implications for data collection, monitoring and cod stock assessments in the Baltic Sea.

## Materials and methods

### Study area

The Baltic Sea is a heavily exploited semi-enclosed brackish-water sea that has experienced substantial changes in anthropogenic pressures and environmental conditions over time [36]. The shallow western Baltic Sea is strongly influenced by well-oxygenated seawater inflows from the Kattegat, while the deeper basins of the southern and eastern Baltic Sea are characterised by strong thermohaline stratification and have been affected by increasing levels of deep-water stagnation and hypoxic bottom conditions [37–39].

The Arkona Sea (SD 24) is a transition area between the shallower Belt Sea (SD 22) and Øresund (SD 23) westwards (around 25 m depth), and the deeper Bornholm Basin (SD 25) eastwards (>90 m), and comprises the Arkona Basin with maximum water depths of 40 to 60 m (Fig 1). The brackish surface water of about 8 psu is separated from the stratified saline bottom water of about 15 to 20 psu by a permanent halocline at a depth of 30 to 40 m [37, 40].

### Sampling

Baltic cod (*Gadus morhua*) samples, i.e. individuals from SD 24 were taken from scientific survey catches between 1977 and 2019 and from commercial catches between 2010 and 2019 (Fig 1 and S1 Fig).

Scientific surveys comprised the German Bottom Trawl Survey between 1977 and 2001 and the German cruises of the Baltic International Trawl survey (BITS) between 2002 and 2019. Both bottom-trawl surveys were mainly directed to demersal fish species, i.e. cod and flatfish, and were conducted twice per year, i.e. in January-March (1st quarter) and October-December (4th quarter; for details refer to S1 Table). Cod samples from German commercial catches originated from active gear (bottom trawl) and passive gear (mainly gillnet) fisheries, which were conducted throughout the year in different locations in SD 24 (Fig 1, S2 Table). The passive gear samples include also a small proportion of cod samples caught with longlines (14%). Longline fisheries took place only in 2015 and 2016 in shallow waters (2 to 16 m, S2 Table).

Cod samples from survey and commercial catches are subsamples of the respective total catch and 10 to 15 individuals per length class per haul were randomly selected for sampling. The samples could not be raised to the length distribution of the respective total catch because length-frequency data were lacking for historical catches.

After capture, cod were measured (total length to the nearest cm), and the sex and maturity stage were determined following [10]. The sagittal otoliths were removed and stored individually in paper bags until further processing.

### Stock discrimination analysis

#### Otolith preparation

For genetic analysis, dried muscle tissue attached to sagittal otoliths was removed using sterile forceps and stored individually in dry 2 ml plastic tubes until further processing. Tissue was attached only to otoliths from the 1970s and 1980s, as otoliths were not cleaned before storage. For otolith shape analysis, we considered only otoliths derived from cod with a total length ≥ 20 cm, as the formation of characteristic lobes of the otolith outline is still incomplete in early life stages of cod [42].

Due to former age reading and archiving procedures, up to 50% of the left and right otoliths from the sampling period between 1977 and 2006 were broken. This occurred particularly often for EBC otoliths, as the otolith annuli of this stock are more difficult to interpret [15]. To avoid a bias, i.e. a high percentage of WBC otoliths and to increase the sample size, all available otolith pieces were glued with a minimal amount of instant glue (UHU all-purpose glue). Successful reassembly was verified under a stereo-microscope by examining the otolith outline for missing edges. Previous studies already reported that gluing of broken cod otoliths has no impact on otolith shape-based discrimination analyses [43]. With this technique, up to 90% of all broken otoliths per sampling year were re-assembled.

#### Otolith shape analysis

Otolith imaging and shape analysis were carried out according to the procedure described in [29]. In brief, high-contrast images of entire and clean cod otoliths were taken with a stereo-microscope (SZX10, Olympus) equipped with a digital microscope camera (Axiocam 105 color, Zeiss). Subsequent shape analyses on otolith images were conducted using normalized elliptic Fourier descriptors from the ShapeR package [44] of R v4.2.1 [45]. The first 12 harmonics (giving 48 shape coefficients) reached 99% of cumulated power percentage and were chosen to describe the shape variations of Baltic cod otoliths.

#### Individual stock assignment

Based on their otolith shape, individuals with unknown stock origin were assigned to either the WBC or EBC stock using a genetically validated baseline with stock-specific shapes derived from [29]. The baseline samples (N = 507) consisted of 52% WBC and 48% EBC individuals and covered a wide range of fish length classes, different sampling areas and years and both sexes to reflect genetic and environmental variations in the otolith shape. As a statistical classifier, a linear discriminant analysis of the baseline samples was used to predict the stock affiliation of individuals with unknown stock origin. The assignment was performed applying the R packages MASS [46] and stats [45]. To estimate the classification accuracy of this approach, we used the shape coefficients of the genetically validated baseline samples and performed a linear discrimination analysis and a leave-one-out cross validation following the procedure described in [29]. This resulted in estimated stock mixing proportions of 51% WBC and 49% EBC. The individual classification accuracy (the percentage of fish correctly assigned to their actual stock) of this approach was 83% [29].

#### Genetic validation of historical cod samples

To confirm that the baseline containing contemporary otolith samples can be used consistently over time for stock discrimination, the stock assignment of a subset of historical cod samples was genetically validated. Genetic analysis of historical samples was run in a laboratory not used for analysis of recent cod samples or other fish samples to prevent potential contamination of historical samples.

Genetic material of historical cod samples was only available from survey catches between 1979 and 1989. DNA was isolated from dried muscle tissue surrounding the otoliths from 314 individuals from SD 24 following the protocol described in [47] and originally developed by [48], with an overnight incubation during the initial lysis step. Samples were genotyped at 38 previously published SNP (single nucleotide polymorphism) loci to unambiguously assign individuals to the WBC or EBC stock following [7]. To detect potentially contaminated DNA extracts, three polymorphic microsatellite loci were amplified (GmoC18: [49]; Tch11 and Tch14: [50]) for each individual in a multiplex PCR reaction using the QIAGEN Multiplex PCR Kit. The reaction volume of 10 μL contained 1 μL of DNA template, 5 μL of QIAGEN Multiplex PCR Master Mix and 0.4 μL of each forward and reverse primer. PCR reaction consisted of an initial denaturation step of 15 min at 95°C followed by 28 cycles of 30s at 94°C, 3 min at 57°C, 60s at 72°C and a final elongation step of 45 min at 60°C. PCR fragments were sized on an ABI 3100 (Applied Biosystems) capillary sequencer and scored using GeneScan Analysis Software. For each locus and individual, the number of alleles was checked to ensure that individuals with no more than two alleles per locus were included in the final dataset for down-stream analysis. Low-quality loci with more than 30% of missing data were omitted from the dataset, leading to a final SNP set of 22 diagnostic SNPs. Additionally, individuals were excluded if > 30% of the genotypes were missing.

For the individual assignment to the WBC or EBC stock, we used genetically assigned reference samples from SD 22 (N = 64; reference WBC) and SD 25 (N = 44; reference EBC) from [7]. Assignment of historical cod samples to the most likely reference stock was based on genotype likelihoods (following [51]) using the programme GeneClass2 [52]. In a second approach, individual assignment at *k* = 2 (number of assumed populations or genetic groups) was conducted using STRUCTURE v.2.3.4 [53] applying the parameters used in [7].

Only individuals with assignment scores ≥ 99% and individuals supported by a combination of GeneClass2 assignment and STRUCTURE analysis were considered as unambiguously assigned samples. A total of 164 individuals passed the sample contamination control based on microsatellite analysis, the missing data threshold and the requirements for stock allocation (S3 Table). Out of these samples, 19 were identified as WBC and 145 were identified as EBC.

To determine the classification success of the otolith shape analysis for the historical cod samples (1979–1989), the agreement between genetic assignment and assignment based on otolith shape was calculated. Individual classification accuracy for historical samples was on average 79%, with 84% of WBC samples and 74% of EBC samples correctly assigned to their original stock based on their otolith shape.

The individual classification accuracy of the otolith shape analysis for historical cod samples (1979–1989) was only slightly lower than for contemporary cod samples (79% versus 83%, respectively), suggesting that the applied approach is a suitable tool to reliably assign historical and contemporary cod samples to their respective stock.

### Data preparation and analysis

For the stock mixing analysis, individual stock assignment was used to calculate total mixing proportions of the two cod stocks, i.e. percentage of WBC and EBC.

To study temporal patterns of stock mixing, a time series of annual mixing proportions of WBC and EBC in SD 24 was developed using cod from scientific trawl catches from 1977 to 2019 (N_Otoliths_ = 20 302), preferably from the 4^th^ quarter. There were only 1^st^ quarter samples available for the years 1984, 1987, 1988 and 1989 and there were no data or archived samples for the years 1980, 1982, 1990 and 1991 (S1 Table). A comparison of mixing proportions based on cod samples from the 1^st^ and 4^th^ quarters (selected years between 1995 and 2016, N_Otoliths_ = 7532) showed only slight differences between quarters within the same calendar year with deviations of 1 to 10% (S2 Fig). This suggests that cod samples from the 1^st^ quarter can also be used as a proxy for the mixing proportions of the 4^th^ quarter within the same calendar year when otoliths from the 4^th^ quarter are missing.

Survey mixing data were separated by sampling decade (1977–1989, 1992–1999, 2000–2009, 2010–2019) and capture depth to also consider temporal effects on depth-specific mixing patterns. For each fishing haul, the mean value of the minimum and maximum capture depth was calculated and categorised into depth strata (0-<10m, 10-<20m, 20-<30m, 30-<40m, 40-<50m, 50-<60m, 60-<70m).

The period with the best spatially resolved sampling (2010–2019, N_Otoliths_ = 6597) was used to investigate also spatial trends in stock mixing. The data were analysed on the level of ICES rectangle; SD 24 is divided into 3 x 3 statistical rectangles of 30 x 30 nautical miles (~55.6 x 55.6 km, Fig 1).

Mixing proportions based on commercial catches covered a sampling period from 2010 to 2019, with gaps for the years 2011, 2013 and 2014 (N_Otoliths_ = 10 924; S2 Table). In all years analysed, mixing proportions varied over the seasons, but no consistent seasonal pattern was evident (S3 Fig). Thus, interannual trends were assessed on the basis of pooled data for each given year to increase sample size within years.

To investigate depth-specific patterns of stock mixing and the stock composition of catches from different fishing fleets, commercial mixing data were grouped by capture depth and sampling year, and by used fishing gear type (active and passive fishing gears).

For survey and commercial data, univariate generalized-linear models (GLM) with Gaussian distribution were fitted to account for spatio-temporal effects on stock mixing using mixing proportions as dependent variable and mean depth, longitude, latitude and capture years of fishing hauls as independent predictors. Only fishing hauls with ≥50 individuals were used in the models. All univariate GLM effects were tested by analysis of deviance, and deviance change was assumed to be approximately χ2-distributed. All statistical analyses were carried out using the R statistical package [45].

## Results

### Spatio-temporal patterns of stock mixing

**Fig 2:**
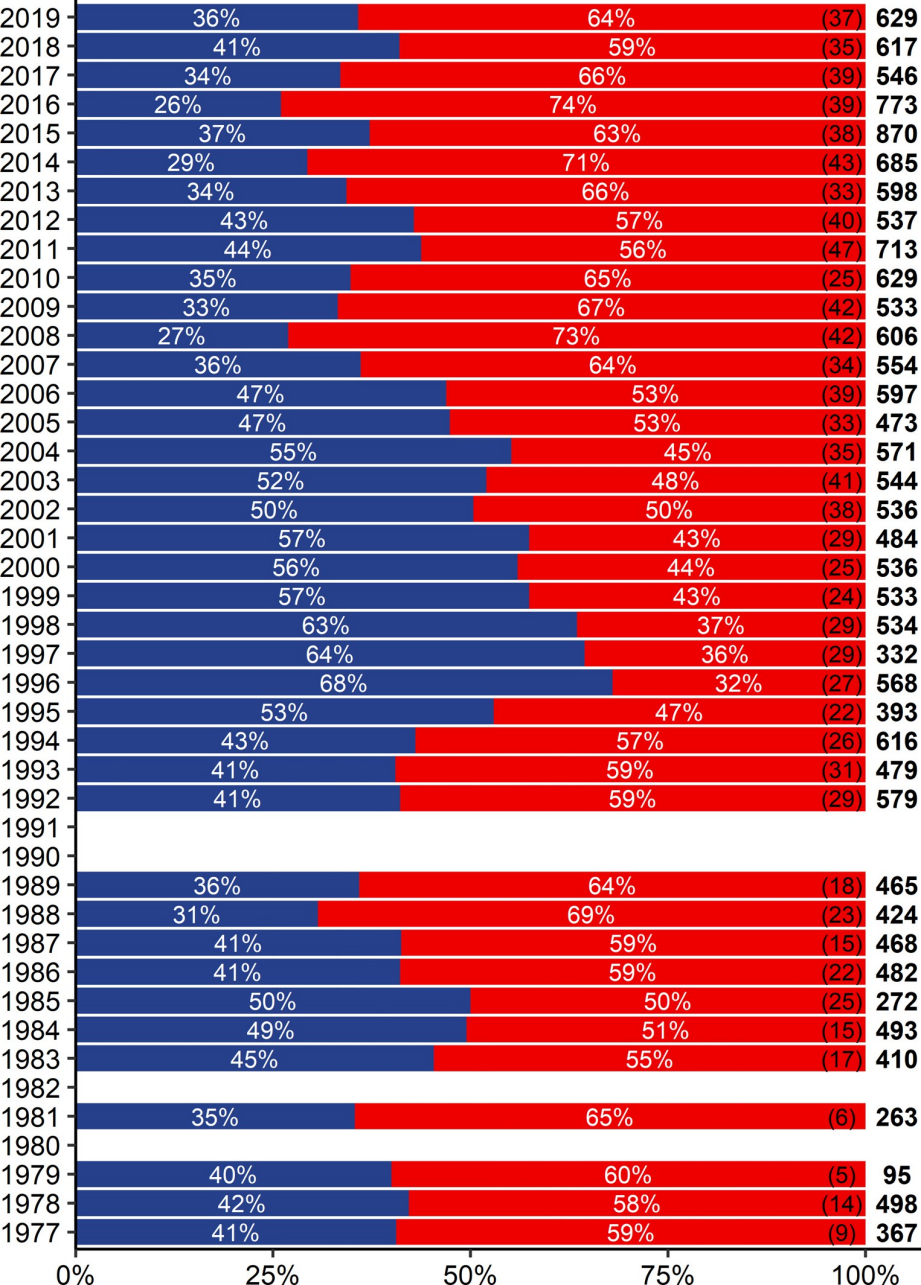
Annual mixing proportions of the Baltic cod stocks in SD 24. Mixing proportions of WBC (blue) and EBC (red) are based on cod samples (N = 20 302) from trawl surveys between 1977 and 2019. Total numbers of fishing hauls used in the stock mixing analysis (in brackets) and absolute numbers of otoliths used in the shape analysis (in bold) are given on the right side of each year. Years without bars = no data available.

The time series of annual stock mixing proportions in SD 24 revealed limited changes in relative stock proportions in the late 1970s and in the 1980s, with an average of 41% WBC and 59% EBC (Fig 2). The proportion of WBC then increased to a maximum of 68% in 1996, before decreasing to 27% in the late 2000s. Over the past ten years, the stock mixing proportions in SD 24 have stabilised again with an average of 36% WBC and 64% EBC. In addition to the temporal trends in stock mixing, survey mixing data of 2010 to 2019 stratified by ICES rectangle showed a clear west-east gradient within SD 24, with the proportion of WBC decreasing from an average of 57% in the western areas, to 39% in the central areas and to 22% in the eastern areas (S4 Fig), which was also reflected in significant effects of sampling longitude on stock mixing proportions (Table 1). The spatial analysis also revealed a weak south-north gradient, with the proportion of WBC decreasing from an average of 48% in the southern areas, to 35% in the central areas and to 30% in the northern part (S4 Fig).

**Table 1:** GLMs containing stock mixing proportions of survey and commercial data as Gaussian response variable and mean depth, longitude, latitude and capture year of fishing hauls as predictors.

Response	Mixing proportions (survey data)					Mixing proportions (commercial data)				
	Df	Dev	ResDf	ResDev	*P*(>Chi)	Df	Dev	ResDf	ResDev	*P*(>Chi)
Null			82	2.618				61	2.777	
Depth	1	0.566	81	2.051	**<0.001**	1	1.812	60	0.965	**<0.001**
Longitude	1	0.169	80	1.882	**<0.001**	1	0.046	59	0.919	0.070
Latitude	1	0.000	79	1.882	0.959	1	0.003	58	0.916	0.655
Year	28	1.254	51	0.629	**<0.001**	6	0.193	52	0.724	**0.032**

Significant values are in bold. Df = Degrees of freedom, Dev = Deviation, ResDf = Residual degrees of freedom, ResDev = Residual deviance.

### Depth-specific patterns of stock mixing over time

**Fig 3:**
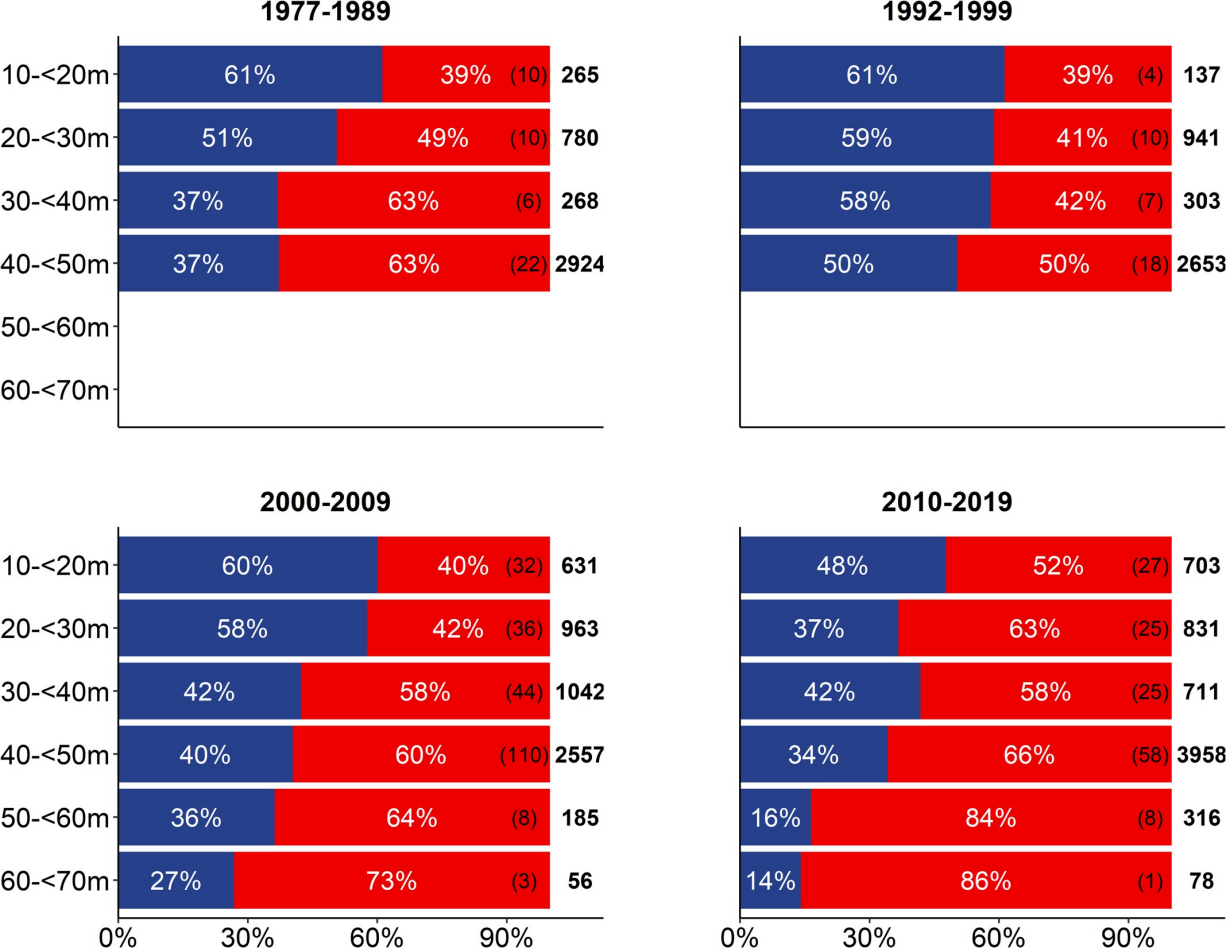
Depth-stratified distribution of stock mixing proportions in SD 24. Mixing proportions of WBC (blue) and EBC (red) are based on cod samples (N = 20 302) from trawl surveys between 1977 and 2019, grouped by capture depth and sampling decade. Total numbers of fishing hauls used in the stock mixing analysis (in brackets) and absolute numbers of otoliths used in the shape analysis (in bold) are given on the right side of each depth stratum. Depths without bars = no data available.

Survey mixing data stratified by capture depth and sampling decade revealed stable depth-specific patterns of stock mixing over time (Fig 3, Table 1), with consistently higher proportions of WBC in shallower compared to intermediate and deeper waters, and a corresponding opposite pattern for EBC. Specifically, the proportion of WBC decreased from an average of 53% in shallower waters (10-<30m depth), to 40% (30-<50m) and to 23% in deeper waters (50-<70m depth). From 2010 to 2019, while relative trends with depth remained the same, the average proportion of WBC was generally lower in shallower and deeper waters (Fig 3), which was also reflected in total proportions of WBC during this sampling decade (Fig 2).

**Fig 4:**
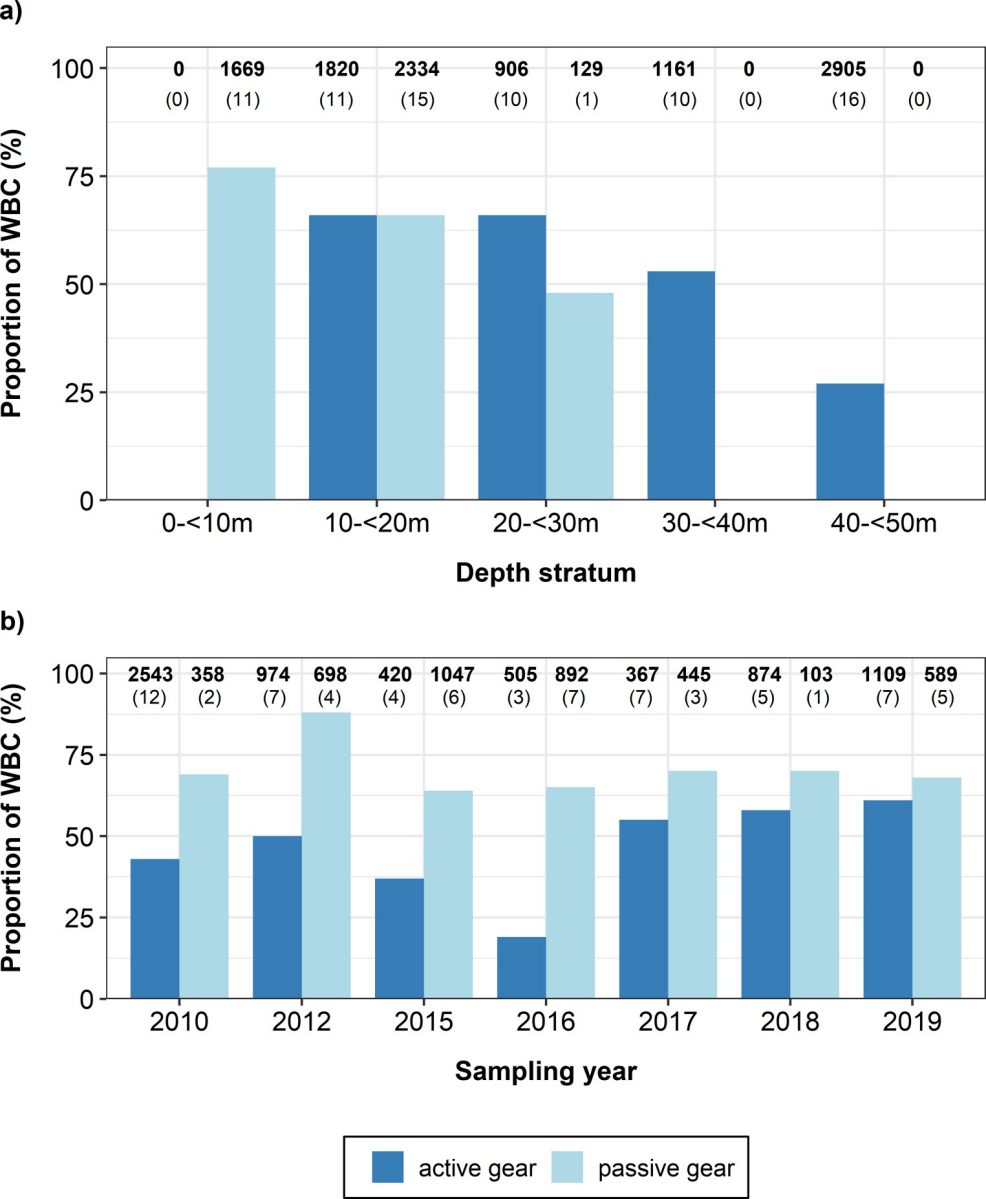
Fishing gear-specific proportions of WBC in SD 24. Mixing proportions are based on cod samples (N = 10 924) from German commercial catches between 2010 and 2019, using different types of fishing gears (active = trawl, passive = mainly gill net). Mixing data were grouped by a) capture depth and b) sampling year. Absolute numbers of otoliths used in the shape analysis (in bold) and total numbers of fishing hauls used in the stock mixing analysis (in brackets) are given on the top of each bar. Depths without bars = no data available.

Stock mixing analyses of cod otoliths from German commercial catches between 2010 and 2019 confirmed depth-specific patterns already revealed by the survey mixing data, with capture depth of fishing hauls as highly significant exploratory variable for the different stock mixing proportions (Table 1). The proportion of WBC decreased from 76% at 0-<10m water depth to 27% at 40-<50m depth (Fig 4A). This result is in line with the depth distribution of the genetically validated baseline samples from SD 24, where the proportion of genetically assigned WBC decreased from 67% at 10-<20m water depth to 14% at 30-<50m depth (S5 Fig).

Commercial mixing data also showed a clear link between the type of fishing gear, capture depth and the proportion of WBC in German waters, with passive fishing gear usually operating in shallower waters catching higher proportions of WBC and active fishing gears in deeper waters with lower proportions of WBC (Fig 4A).

### Fishing gear-specific exploitation of different stocks

Commercial mixing data stratified by gear type and sampling year detected different mixing proportions of WBC and EBC between active and passive gear fisheries in SD 24 (Fig 4B). Higher proportions of WBC were found in catches using passive fishing gears (on average 71%, ranging from 64 to 88%), while lower proportions of WBC occurred in catches using active fishing gears (on average 46%, ranging from 19 to 61%). The high proportions of WBC in passive gears catches were consistent irrespective of the fishing gear (i.e. gillnets or longlines). Estimated mixing proportions based on commercial cod samples from active gear fisheries were generally in line with the estimates based on survey cod sampled during scientific trawl surveys (Fig 2).

**Fig 5:**
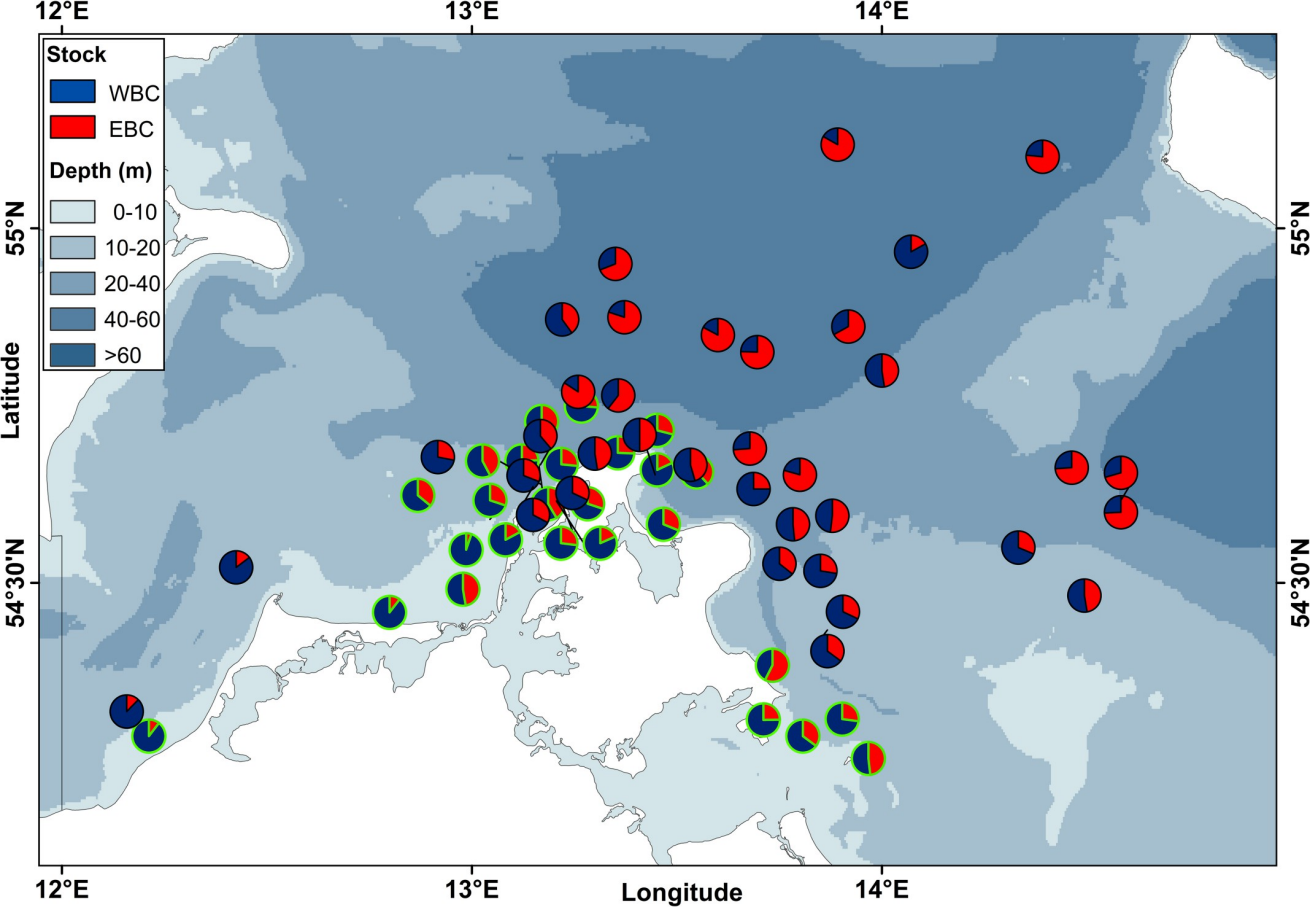
Spatial distribution of stock mixing proportions in SD 24. Mixing proportions of WBC and EBC are based on cod samples from German commercial catches between 2010 and 2019. Samples from active and passive gear fisheries are marked with black and green outlines, respectively. Only fishing hauls with ≥50 individuals are shown (N_total_ = 10 631 otoliths from 62 sampled hauls).

Independent from the type of fishing gear, spatial analysis of commercial mixing data showed a clear increasing trend of WBC towards shallower waters and towards the coastline in the western Arkona Sea (Fig 5).

## Discussion

### Spatio-temporal patterns of stock mixing

In the present study, long-term stock mixing data of Baltic cod confirmed that WBC and EBC coexist in the Arkona Sea, but over a much longer time period than previously assessed by other studies [8, 28] and with fluctuating mixing proportions over the 43-year observation period (1977–2019).

Over the last five decades, the cod stocks permanently mixed in the Arkona Sea and no stock superseded the other stock from SD 24. However, there is a strong correlation between stock size (i.e. SSB) and stock mixing proportions (S6 Fig). If the SSB of both stocks is either high or low, mixing ratios remain stable; if the SSB of only one stock changes, mixing ratios also change. For instance, the SSB of WBC and EBC was at a record high in the 1980s [6] and mixing proportions revealed in this study were relatively stable in SD 24 in the late 1970s and 1980s (~59% EBC, S6 Fig). In the 1990s, only the stock size of EBC declined drastically [6], which resulted in lower EBC percentages in SD 24 (minimum of 32% in 1996, S6 Fig). This shift is in line with the estimated mixing proportions of ^~^ 30–40% EBC for 1996 and 1998 by [28]. It is assumed that the decrease in stock size of EBC in the 1990s was caused by a strong increase in fishing effort and unfavourable conditions for recruitment [32].

The EBC stock slightly recovered in the 2000s, while the SSB of WBC decreased, leading to increasing percentages of EBC in the Arkona Sea (a maximum of 73% in 2008, S6 Fig). This trend was also observed in [28], with EBC percentages of ~70% in the late 2000s. However, the recovering EBC stock did not re-colonize their former north-easterly territories (i.e. Gotland basin; [54]). The spreading of areas with hypoxic or even anoxic conditions in the Gotland basin resulted in a large-scale loss of historic spawning grounds and hence a massive decrease in cod abundance in the northern area [34]. As a consequence, EBC is presently mainly concentrated in the southern Baltic Sea (SDs 25 and 26) and in SD 24 [34, 35].

In the last 10 years, the average stock mixing proportions of cod in SD 24 were relatively stable (~64% EBC), as the SSB of both stocks strongly declined to historic lows (S6 Fig; [6, 35]). Furthermore, the eastern and western parts of SD 24 are used by significant proportions of WBC (on average 30%) and EBC (on average 40%), respectively, which suggests that stock mixing of cod may not be restricted to the artificial boundaries of SD 24. Hence, future stock mixing analysis of Baltic cod may also consider assessing the stock mixing beyond the boundaries of SD 24 to improve the understanding of the mixing dynamics.

In general, ambient environmental conditions may have an impact on annual stock mixing proportions in SD 24, albeit these interactions might be difficult to investigate. Environmental parameters, such as water temperatures, salinity and oxygen levels, are highly variable within the mixing area, and mean values for these abiotic factors might not be representative for the environmental conditions in the Arkona Sea. A recent study suggested that fluctuating environmental conditions within the Arkona Sea are likely to have a rather small effect on stock mixing of Baltic cod, as they found no significant interactions between mixing proportions and examined environmental parameters within SD 24 [8]. It is assumed that variations in stock mixing proportions may be rather associated with environmental conditions beyond SD 24.

### Shallow-water WBC and deep-water EBC

Depth-stratified mixing analysis revealed a strong correlation between capture depth and stock mixing patterns, with higher proportions of WBC in shallower waters and coastal habitats and increasing proportions of EBC in deeper waters and offshore habitats. Depth-specific mixing patterns were consistent between commercial and survey data, indicating that the long-term coexistence of Baltic cod stocks in SD 24 might be attributed to differences in depth zonation of WBC and EBC.

Basin-specific hydrographical conditions in the Baltic Sea provide diverging spawning and feeding environments for the same species in different areas. The core area of WBC are the shallow-waters of the western Baltic Sea, characterized by a permanent halocline and a mixed water column during winter. WBC display pronounced seasonal changes in depth distribution and use very shallow nearshore waters in spring and autumn, and deeper waters in summer (avoidance of warmed surface waters) and winter (for spawning; [55]), however mostly within a depth range of about 25 m because SD 22 and SD 23 are relatively shallow coastal areas. WBC experience regularly high water temperatures in summer, but hardly any hypoxia [56]. The bulk of EBC occupy the deeper basins of the eastern Baltic Sea, with a permanent stratification in terms of salinity, temperature and oxygen. EBC use the extensive slope areas and, at times when hypoxia restricts bottom access, also the pelagic zone. Overall, EBC encounter lower salinities and regularly hypoxia [57, 58]. The depth-specific differences in stock mixing proportions observed in the Arkona Sea reflect the general habitat use of WBC and EBC in their core distributions in the Baltic Sea. WBC are apparently adapted to forage in the shallow-water areas in SD 22 and SD 23 and the zone above the halocline in SD 24. In contrast, EBC are adapted to forage along the vast slope areas of the deeper eastern Baltic basins and dominate the cod catches in the area below the halocline in SD 24. Thus, a relatively loose habitat segregation may be the mechanism promoting the coexistence of the two cod stocks in SD 24.

Similar depth-specific behavioural patterns were also reported for several other coexisting cod stocks in the North Atlantic, e.g. Norwegian coastal cod and Northeast Arctic cod, which display stationary and migratory behaviours, respectively [19, 59]. Differences in depth distribution and habitat use have also been observed for Icelandic cod populations [20, 22], for coastal cod in southern Norway and off-shore North Sea cod [1, 60] and for inshore and offshore cod off Newfoundland [61]. This suggests that colonisation of different depth zones is a successful life-history strategy facilitating stable coexistence of cod populations.

In fact, several adaptations to life in shallow-water and deep-water habitats have already been found in WBC and EBC, respectively. For instance, otolith characteristics and the length-girth relationship differ between cod stocks. EBC have narrower otoliths [23, 29] and a more streamlined body shape [62, 63], which may be attributed to the differences in the foraging and movement behaviour of the cod stocks. Other studies showed similar adaptations on otolith and body morphology in migratory cod [22, 64, 65], suggesting that a more streamlined otolith and body shape is advantageous for foraging in deeper waters and more active swimming behaviour.

Another morphological trait that may be related to lower temperatures in the deeper basins is the number of vertebrae, which is significantly higher for EBC (reviewed by [57]). Cold water temperatures during egg and larval development have led to a prolonged larval period and an increased number of vertebrae for cod in the western Atlantic [66]. However, a higher number of vertebrae may also be beneficial for vertical movements, as migratory cod off Norway, in the Gulf of St. Lawrence and on the Scotian Shelf [67, 68] have more vertebrae compared to stationary cod populations.

Divergent environmental conditions in the main spawning and feeding habitats of the Baltic cod stocks also provoked physiological modifications. EBC produce larger and lighter eggs that reach neutral buoyancy at a lower salinity level than those of WBC [69]. The expression of a haemoglobin genotype with high oxygen-affinity enables EBC also to better cope with the hypoxic conditions in the deeper basins of the eastern Baltic Sea [70, 71].

In addition, vertical movement behaviour was related to the Pantophysin I locus of cod populations, with individuals carrying the *Pan* I^AA^ genotype preferring shallow-water habitats, while cod carrying the *Pan* I^BB^ genotype were more often found in deeper waters [72–74]. However, the underlying genetic mechanism is still only poorly understood and direct evidence for the connection between cod movement behaviour types and the role played by *Pan* I is still lacking.

### Fishing gear-induced bias in the stock assessments

Stock mixing analysis of cod from commercial catches detected considerably different mixing proportions between active and passive fishing gears, with higher proportions of EBC caught with bottom trawls in deeper waters and higher proportions of WBC caught mainly with gillnets in shallower waters. In German waters, specific depth zones are exploited by different fishing gears, i.e. the active gear fleet usually operates offshore in waters deeper than 20 m, while the passive gear fleet uses nearshore waters shallower than 20 m to avoid disturbance by trawlers [55, 75]. Due to the differences in depth distribution and habitat use of WBC and EBC, catches from different fleets result in different mixing ratios of Baltic cod stocks and whether or not this is considered in data collection may influence the quality of the mixing data and the stock assessments.

In the present Baltic cod stock assessments, samples used to evaluate the stock status and to quantify stock mixing originate from scientific surveys and commercial catches using only active fishing gears [35]. Findings of this study clearly show that WBC favour shallow-water habitats in the Arkona Sea (SD 24), suggesting that trawl surveys underestimate the proportion of WBC in SD 24, particularly in the 4^th^ quarter, when WBC intensively use shallow-water areas for feeding [55]. Present estimates of SSB and stock mixing might be biased because mixing proportions of trawl samples from deeper stations are extrapolated to catches from shallower waters, mainly originating from passive gears. This bias may induce uncertainties in the current stock assessments but since the present contribution of passive gear catches in SD 24 is low, and given a lack of mixing proportions from the passive gear fisheries of other countries, the bias is considered acceptable. However, thoroughly accounting for the natural distribution of Baltic cod stocks in sampling and analyses of commercial and fisheries-independent catches is likely to improve our understanding of Baltic cod ecology and increase the quality of data used in the stock assessments.

## Conclusions

The combined approach of a genetically validated baseline and otolith shape analysis enabled the successful reconstruction of a 43-year time series of mixing proportions of WBC and EBC in an important fishing area of the Baltic Sea (SD 24). The present study confirmed the coexistence of the cod stocks in the Arkona Sea, albeit with fluctuating mixing proportions over the last five decades, which were potentially driven by changes in stock sizes. The study revealed a strong correlation between capture depth and stock mixing patterns, with higher proportions of WBC in shallower waters and more coastal habitats and increasing proportions of EBC in deeper waters and more offshore habitats. Consistent depth-specific mixing patterns indicate stable differences in depth distribution and habitat use of the cod stocks, which may be the underlying mechanism promoting the long-term coexistence of the two stocks in SD 24. These differences were also reflected in significantly different proportions of WBC and EBC in fisheries applying passive gears in shallower waters (more WBC) and active gears in deeper waters (more EBC). This highlights the potential for fishing gear-specific exploitation of different stocks and calls for stronger consideration of capture depth and gear type in stock assessments. These novel insights provide the basis for improved approaches to research, monitoring and management of Baltic cod stocks.

## Supporting information

S1 Fig Sampling locations of Baltic cod in SD 24 separated by sampling decade. Cod samples originate from bottom-trawl survey catches between 1977 and 2019.
(TIF)

S2 Fig Proportion of WBC in SD 24. Mixing proportions are based on cod samples from 1^st^ and 4^th^ quarter trawl surveys between 1995 and 2016 (selected years, N_Otoliths_ = 7532). Absolute numbers of otoliths used in the shape analysis are given on the top of each bar.
(TIF)

S3 Fig Seasonal proportions of WBC in SD 24. Mixing proportions are based on cod samples from German commercial catches between 2010 and 2019 (selected years, N_Otoliths_ = 10 924). Active and passive gear samples are pooled. Absolute numbers of otoliths used in the shape analysis are given on the top of each bar. Quarters without bars = no data available.
(TIF)

S4 Fig Spatial distribution of annual stock mixing proportions in SD 24. Mixing proportions of WBC (blue) and EBC (red) are based on cod samples (N = 6597) from trawl surveys between 2010 and 2019, grouped by ICES rectangles (see Fig 1 for statistical rectangles). Rectangles are arranged according to their relative position within SD 24 from west to east and from north to south (Fig 1). Absolute numbers of otoliths used in the shape analysis are given on the right side of each year. Years without bars = no data available.
(TIF)

S5 Fig Proportion of genetically identified WBC in SD 24. Mixing proportions are based on genetically validated baseline samples (N = 348) from [29]. Mixing data were grouped by capture depth and used type of fishing gear (active = trawl, passive = mainly gillnet). Absolute numbers of cod samples used for genetic analysis (in bold) and total numbers of fishing hauls used in the stock mixing analysis (in brackets) are given on the top of each bar. Depths without bars = no data available.
(TIF)

S6 Fig Mixing proportions and spawning stock biomass of Baltic cod stocks. a) Annual mixing proportions of EBC in SD 24 based on cod otoliths (N = 20 302) from trawl surveys between 1977 and 2019 (black bars) and annual proportions of spawning stock biomass (SSB) of EBC on total cod SSB (i.e. SSB_EBC+_SSB_WBC_) in the Baltic Sea (red line). b) Correlation analysis of proportion of EBC in SD 24 and proportion of SSB of EBC in the Baltic Sea. WBC = Western Baltic Cod, R = Pearson correlation coefficient.
(TIF)

S1 Table Summary of cod samples from German trawl survey catches in SD 24 between 1977 and 2019. Table comprises sampling year and months, sample size (N), sampled areas (A = 12°-13° E, B = 13°-14° E, C = 14°-15° E), total fish length (range and mean ± SD (standard deviation)), proportion of spawning individuals (maturity stage 5 and 6, following [10]) and proportion of female fish. *These samples were used only for the comparison of mixing proportions between quarters within the same year.
(DOCX)

S2 Table Summary of cod samples from German commercial catches in SD 24 between 2005 and 2019. Definition of table headers and items as in S1 Table. Fishing gear: active = trawl, passive = gillnet. *In 2015 and 2016, 284 and 296 cod were caught with longlines, respectively.
(DOCX)

S3 Table Overview of historical cod samples from German trawl survey catches in SD 24 between 1979 and 1989 used for genetic analysis. Definition of table headers and items as in S1 Table.
(DOCX)
