# Supplementary figures and images for: Living apart together: Long-term coexistence of Baltic cod stocks associated with depth-specific habitat use
Source: PLoS One. 2022 Sep 28;17(9):e0274476. doi: 10.1371/journal.pone.0274476 (PMC9518848; doi:10.1371/journal.pone.0274476)

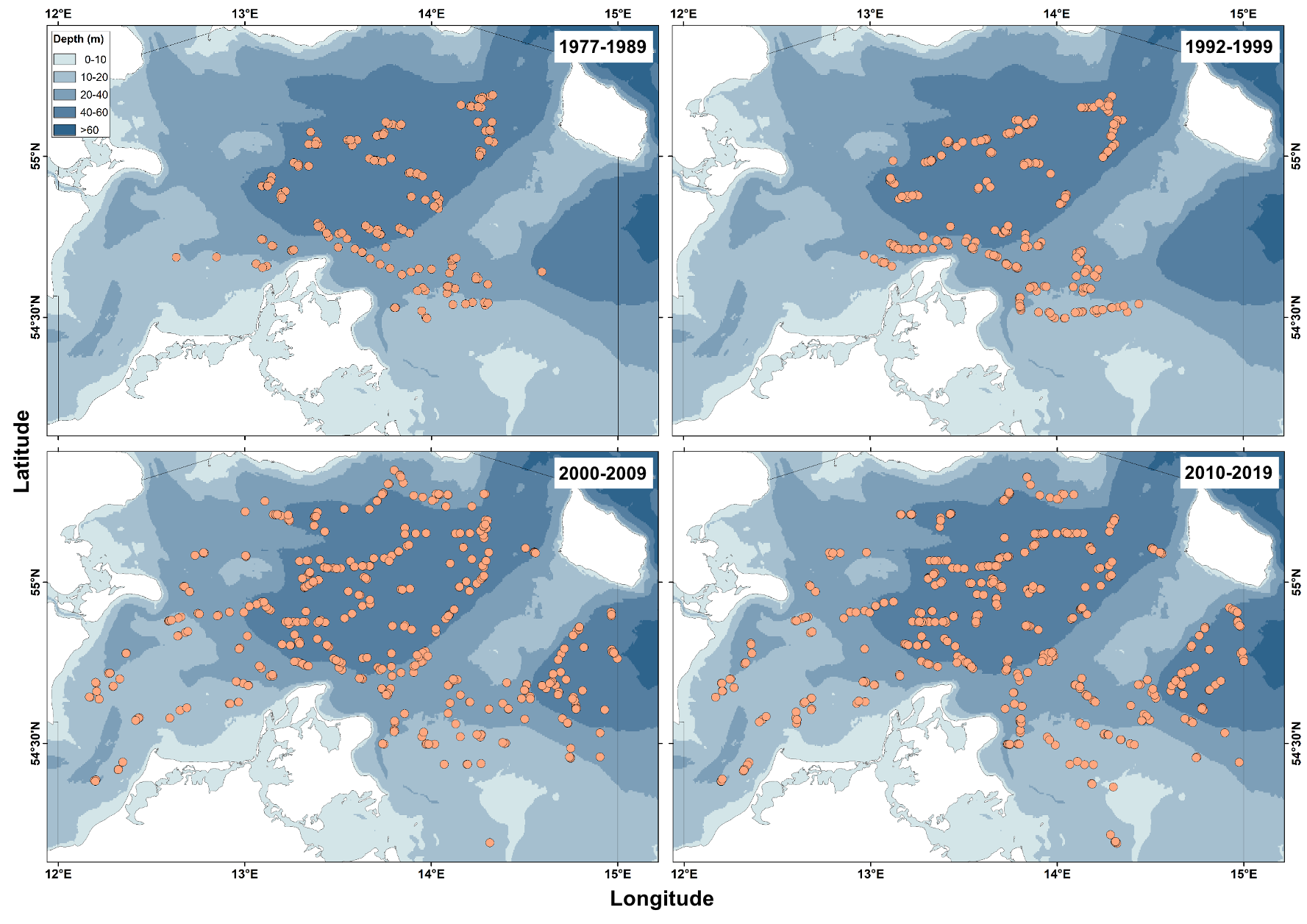

Supplement: S1 Fig — Cod samples originate from bottom-trawl survey catches between 1977 and 2019. (TIF) [file pone.0274476.s001.tif]

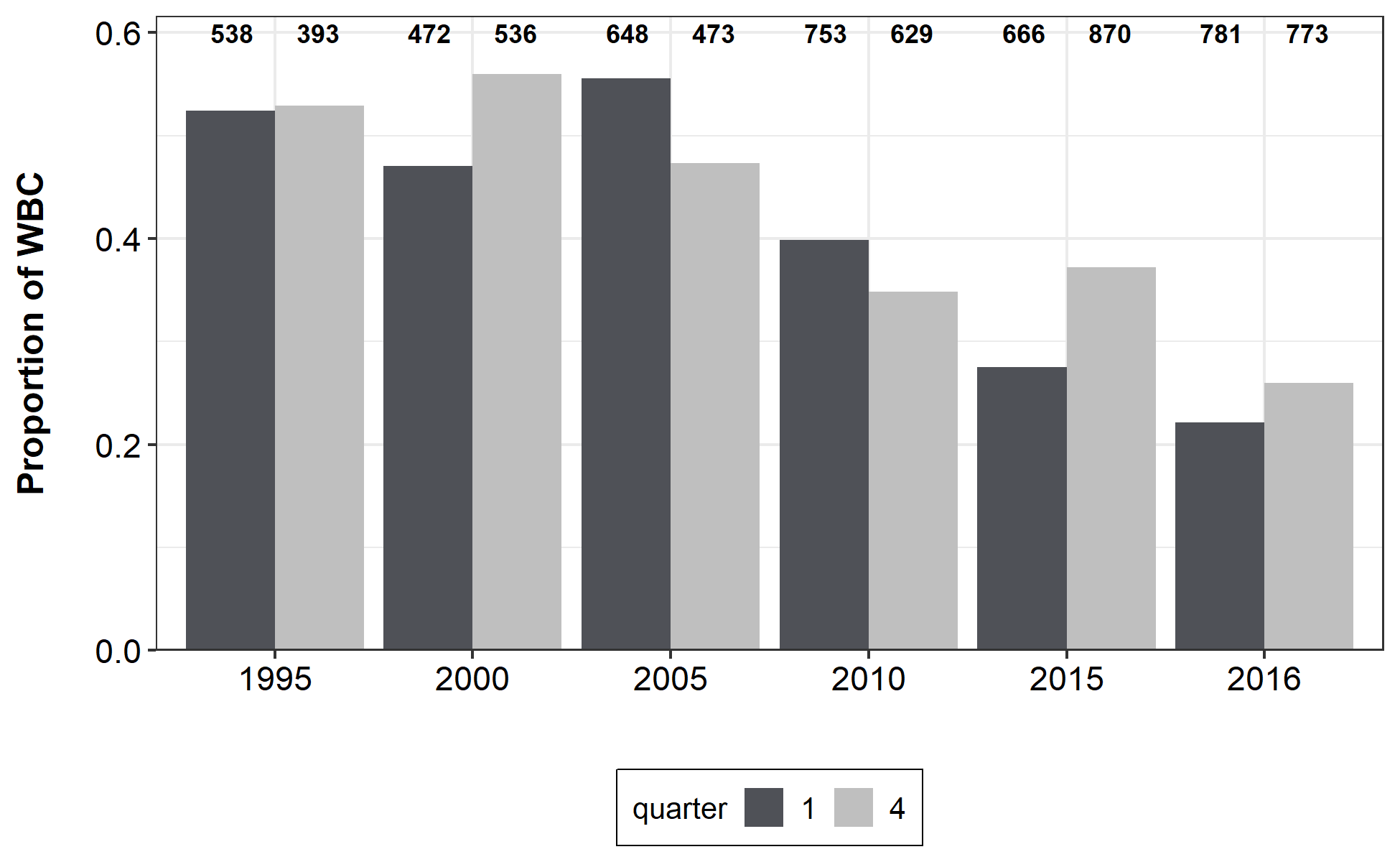

Supplement: S2 Fig — Mixing proportions are based on cod samples from 1st and 4th quarter trawl surveys between 1995 and 2016 (selected years, NOtoliths = 7532). Absolute numbers of otoliths used in the shape analysis are given on the top of each bar. (TIF) [file pone.0274476.s002.tif]

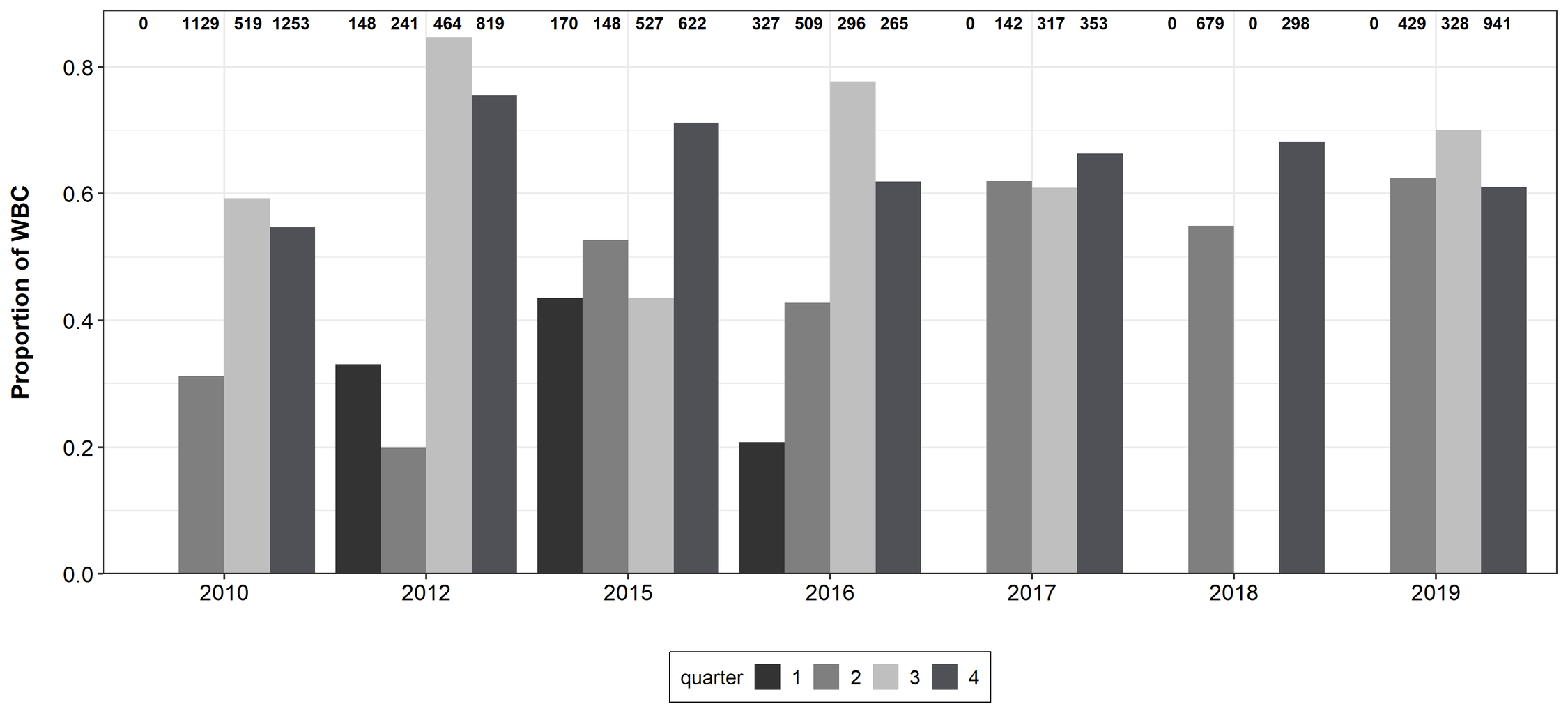

Supplement: S3 Fig — Mixing proportions are based on cod samples from German commercial catches between 2010 and 2019 (selected years, NOtoliths = 10 924). Active and passive gear samples are pooled. Absolute numbers of otoliths used in the shape analysis are given on the top of each bar. Quarters without bars = no data available. (TIF) [file pone.0274476.s003.tif]

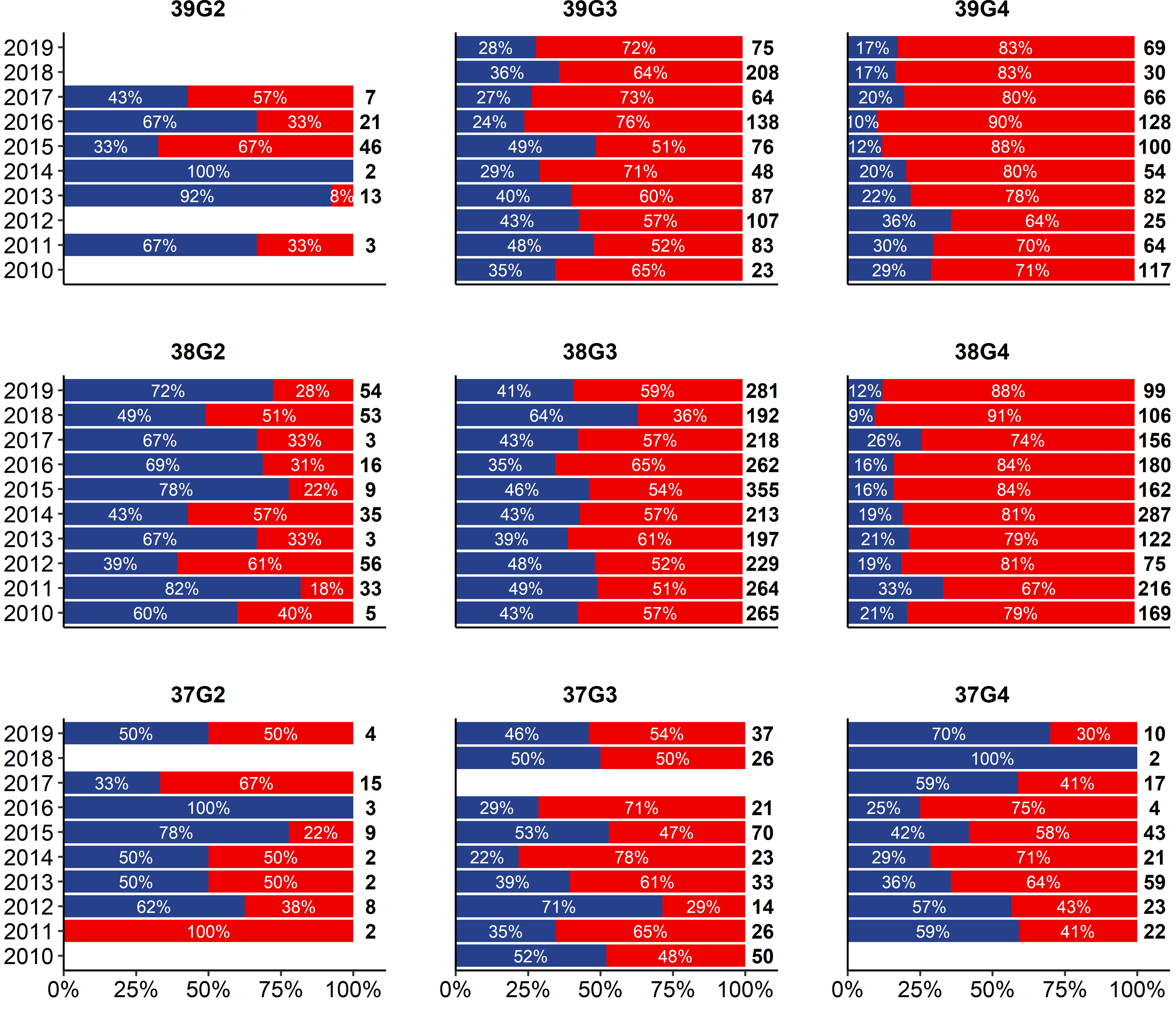

Supplement: S4 Fig — Mixing proportions of WBC (blue) and EBC (red) are based on cod samples (N = 6597) from trawl surveys between 2010 and 2019, grouped by ICES rectangles (see Fig 1 for statistical rectangles). Rectangles are arranged according to their relative position within SD 24 from west to east and from north to south (Fig 1). Absolute numbers of otoliths used in the shape analysis are given on the right side of each year. Years without bars = no data available. (TIF) [file pone.0274476.s004.tif]

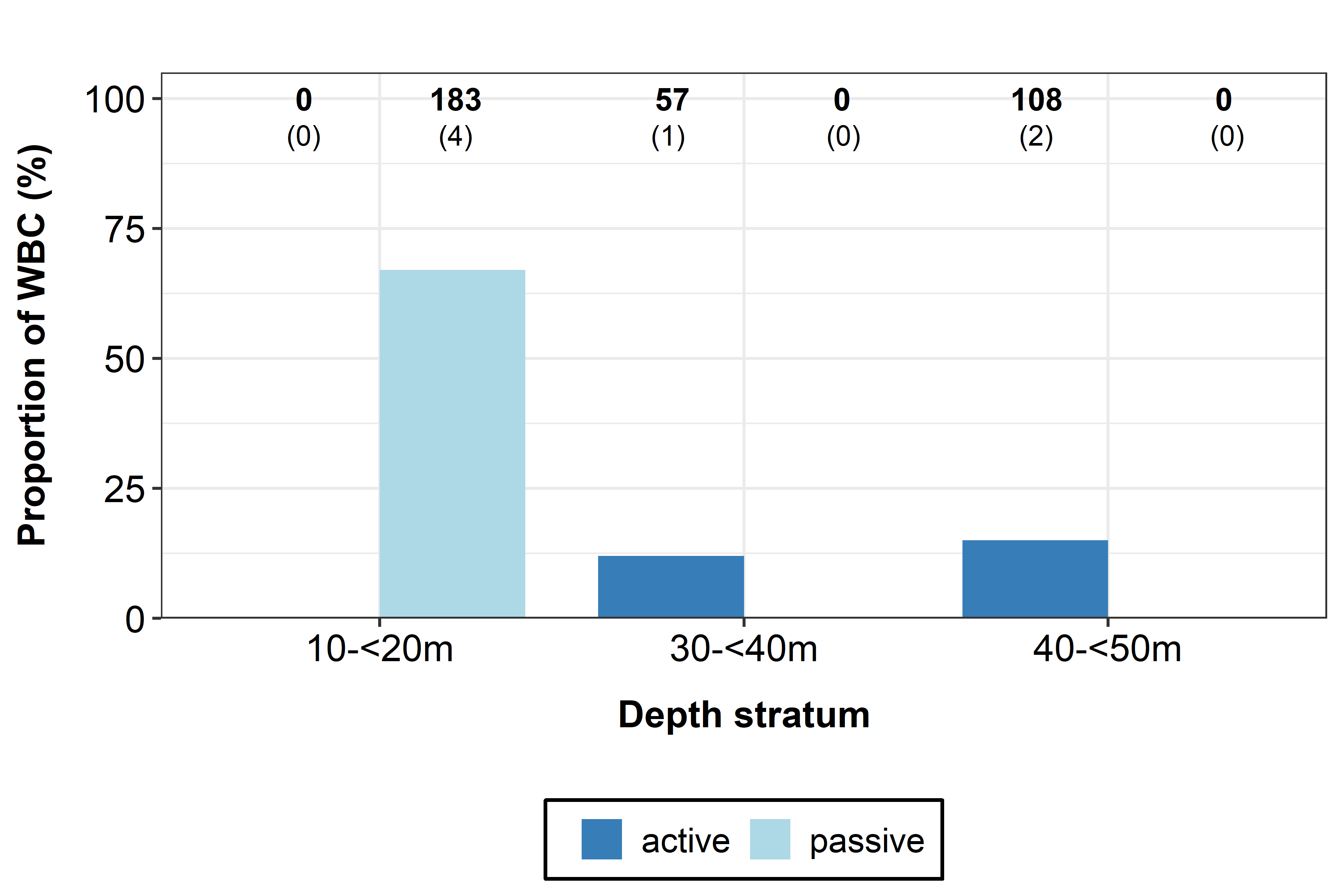

Supplement: S5 Fig — Mixing proportions are based on genetically validated baseline samples (N = 348) from [29]. Mixing data were grouped by capture depth and used type of fishing gear (active = trawl, passive = mainly gillnet). Absolute numbers of cod samples used for genetic analysis (in bold) and total numbers of fishing hauls used in the stock mixing analysis (in brackets) are given on the top of each bar. Depths without bars = no data available. (TIF) [file pone.0274476.s005.tif]

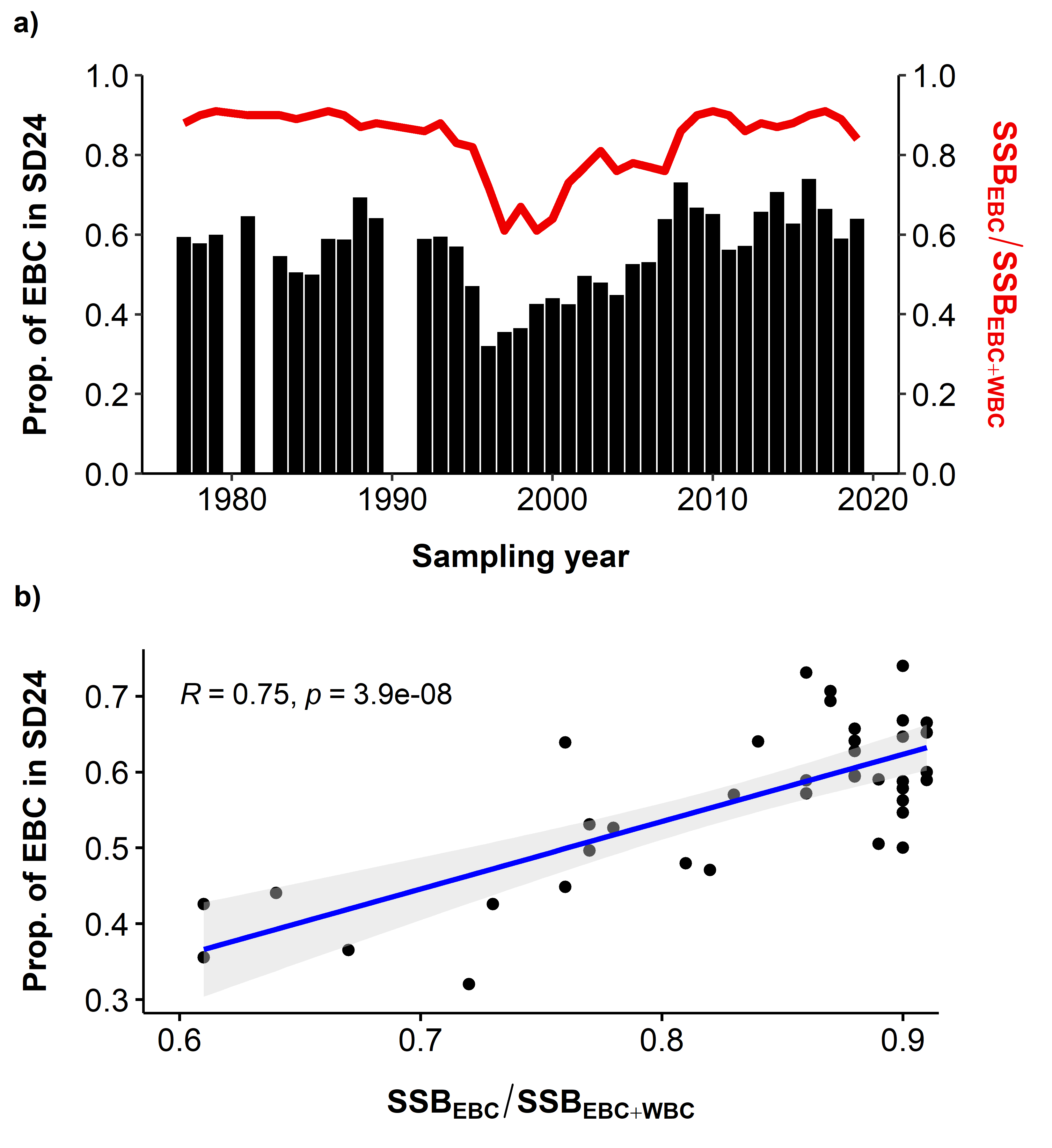

Supplement: S6 Fig — a) Annual mixing proportions of EBC in SD 24 based on cod otoliths (N = 20 302) from trawl surveys between 1977 and 2019 (black bars) and annual proportions of spawning stock biomass (SSB) of EBC on total cod SSB (i.e. SSBEBC+SSBWBC) in the Baltic Sea (red line). b) Correlation analysis of proportion of EBC in SD 24 and proportion of SSB of EBC in the Baltic Sea. WBC = Western Baltic Cod, R = Pearson correlation coefficient. (TIF) [file pone.0274476.s006.tif]
