# Supplementary material for: Living apart together: Long-term coexistence of Baltic cod stocks associated with depth-specific habitat use
Source: PLoS One. 2022 Sep 28;17(9):e0274476. doi: 10.1371/journal.pone.0274476 (PMC9518848; doi:10.1371/journal.pone.0274476)
Supplement: S1 Table — Table comprises sampling year and months, sample size (N), sampled areas (A = 12°-13° E, B = 13°-14° E, C = 14°-15° E), total fish length (range and mean ± SD (standard deviation)), proportion of spawning individuals (maturity stage 5 and 6, following [10]) and proportion of female fish. *These samples were used only for the comparison of mixing proportions between quarters within the same year. (DOCX) [file pone.0274476.s007.docx]

| **Year/month(s)** | **N** | **Sampled areas** | **Length range [cm]** | **Mean length ± SD [cm]** | **Spawning fish [%]** | **Female fish [%]** |
| --- | --- | --- | --- | --- | --- | --- |
| 1977/11 | 367 | A,B,C | 20-56 | 30.7 ± 6.3 | 0 | 52.3 |
| 1978/11 | 498 | B,C | 20-79 | 37.9 ± 11.2 | 0 | 49.2 |
| 1979/11 | 95 | B,C | 20-69 | 42.9 ± 13.2 | 0 | 49.5 |
| 1981/11 | 263 | B,C | 20-102 | 38.1 ± 11.5 | 0 | 56.4 |
| 1983/12 | 410 | B,C | 20-115 | 39.3 ± 11.9 | 0 | 54.9 |
| 1984/1 | 493 | B,C | 20-89 | 40.6 ± 12.8 | 0 | 55.6 |
| 1985/12 | 272 | B,C | 20-88 | 46.6 ± 16.7 | 0 | 63.6 |
| 1986/12 | 482 | B,C | 20-66 | 39.2 ± 9.6 | <1 | 48.8 |
| 1987/1+2 | 468 | B,C | 20-81 | 40.9 ± 11.5 | 4.7 | 46.6 |
| 1988/2 | 424 | B,C | 20-72 | 38.2 ± 8.9 | 7.5 | 51.9 |
| 1989/1+2 | 465 | B,C | 20-89 | 44.8 ± 9.2 | 3.0 | 48.2 |
| 1992/11 | 579 | B,C | 20-96 | 36.6 ± 10.8 | 0 | 50.6 |
| 1993/11+12 | 479 | B,C | 20-78 | 39.6 ± 10.0 | 0 | 50.3 |
| 1994/11 | 616 | B,C | 20-107 | 38.8 ± 10.6 | 0 | 55.2 |
| 1995/2* | 538 | B,C | 20-95 | 38.7 ± 15.1 | <1 | 56.1 |
| 1995/11 | 393 | B,C | 20-80 | 42.5 ± 12.1 | <1 | 55.5 |
| 1996/11 | 568 | B,C | 20-103 | 41.3 ± 11.0 | <1 | 48.1 |
| 1997/11 | 332 | B,C | 20-71 | 37.4± 12.1 | 0 | 46.4 |
| 1998/11 | 534 | B,C | 20-95 | 40.5 ± 12.1 | 0 | 54.5 |
| 1999/11 | 533 | A,B,C | 20-72 | 38.3 ± 10.4 | 0 | 57.6 |
| 2000/2* | 472 | A,B,C | 20-91 | 35.5 ± 11.6 | 2.8 | 55.3 |
| 2000/11 | 536 | B,C | 20-91 | 38.4 ± 10.5 | 0 | 58.4 |
| 2001/11 | 484 | A,B,C | 20-80 | 39.2 ± 10.1 | 0 | 51.9 |
| 2002/11+12 | 536 | A,B,C | 20-99 | 38.8 ± 11.7 | 0 | 53.9 |
| 2003/11+12 | 544 | A,B,C | 20-98 | 37.0 ± 9.5 | <1 | 52.0 |
| 2004/10+11 | 571 | A,B,C | 20-79 | 37.8 ± 10.6 | 0 | 50.1 |
| 2005/2* | 648 | A,B,C | 20-102 | 40.5 ± 12.6 | 2.2 | 55.1 |
| 2005/11 | 473 | A,B,C | 20-80 | 40.5 ± 12.3 | 0 | 49.7 |
| 2006/11 | 597 | A,B,C | 20-84 | 40.7 ± 12.4 | 0 | 53.3 |
| 2007/11 | 554 | A,B,C | 20-101 | 39.9 ± 12.1 | <1 | 52.5 |
| 2008/10+11 | 606 | A,B,C | 20-89 | 40.9 ± 12.3 | 0 | 54.8 |
| 2009/10+11 | 533 | A,B,C | 20-92 | 40.7 ± 11.5 | 0 | 51.6 |
| 2010/2* | 753 | A,B,C | 20-104 | 42.7 ± 13.3 | 5.3 | 57.1 |
| 2010/11 | 629 | A,B,C | 20-78 | 40.2 ± 10.8 | <1 | 53.9 |
| 2011/10+11 | 713 | A,B,C | 20-77 | 40.9 ± 11.4 | <1 | 55.8 |
| 2012/10+11 | 537 | A,B,C | 20-75 | 38.7 ± 10.5 | 0 | 49.0 |
| 2013/10+11 | 598 | A,B,C | 20-87 | 36.7 ± 10.2 | 0 | 53.2 |
| 2014/10+11 | 685 | A,B,C | 20-74 | 37.7 ± 9.3 | <1 | 55.5 |
| 2015/2+3* | 666 | A,B,C | 20-74 | 39.5 ± 10.0 | 5.2 | 64.0 |
| 2015/11 | 870 | A,B,C | 20-82 | 40.0 ± 10.7 | <1 | 52.5 |
| 2016/2* | 781 | A,B,C | 20-63 | 37.8 ± 9.3 | 5.8 | 54.8 |
| 2016/11 | 773 | A,B,C | 20-88 | 38.4 ± 10.3 | 0 | 48.4 |
| 2017/11 | 546 | A,B,C | 20-76 | 38.1 ± 9.8 | <1 | 47.3 |
| 2018/11 | 617 | A,B,C | 20-63 | 38.8 ± 9.9 | <1 | 51.2 |
| 2019/11 | 629 | A,B,C | 20-86 | 40.0 ± 10.8 | 1.4 | 48.2 |
