# Supplementary material for: Living apart together: Long-term coexistence of Baltic cod stocks associated with depth-specific habitat use
Source: PLoS One. 2022 Sep 28;17(9):e0274476. doi: 10.1371/journal.pone.0274476 (PMC9518848; doi:10.1371/journal.pone.0274476)
Supplement: S2 Table — Definition of table headers and items as in S1 Table. Fishing gear: active = trawl, passive = gillnet. *In 2015 and 2016, 284 and 296 cod were caught with longlines, respectively. (DOCX) [file pone.0274476.s008.docx]

| **Year** | **Quarters** | **N** | **Sampled areas** | **Length range [cm]** | **Mean length ± SD [cm]** | **Spawning fish [%]** | **Female fish [%]** | **Fishing gear** |
| --- | --- | --- | --- | --- | --- | --- | --- | --- |
| 2010 | 2-4 | 2543 | A,B,C | 20-92 | 44.0 ± 8.0 | 9.8 | 59.6 | active |
| 2010 | 4 | 358 | B | 22-63 | 45.7 ± 5.4 | 0 | 62.6 | passive |
| 2012 | 1-4 | 974 | B,C | 20-73 | 44.6 ± 7.7 | 19.4 | 59.0 | active |
| 2012 | 3,4 | 698 | A,B | 25-73 | 49.6 ± 5.9 | <1 | 63.2 | passive |
| 2015 | 1,3,4 | 420 | B,C | 24-67 | 41.5 ± 7.9 | <1 | 63.8 | active |
| 2015* | 1-4 | 1047 | A,B | 21-65 | 43.5 ± 7.8 | <1 | 64.3 | passive |
| 2016 | 1,2 | 505 | B | 24-69 | 39.3 ± 5.9 | 11.5 | 50.1 | active |
| 2016* | 2-4 | 892 | A,B,C | 23-76 | 46.3 ± 7.8 | 1.9 | 62.8 | passive |
| 2017 | 3,4 | 367 | B | 20-59 | 28.7 ± 7.8 | <1 | 51.2 | active |
| 2017 | 2-4 | 445 | B | 22-86 | 46.8 ± 13.1 | 2.2 | 56.6 | passive |
| 2018 | 2,4 | 874 | A,B,C | 21-87 | 40.4 ± 7.8 | 11.3 | 51.3 | active |
| 2018 | 2 | 103 | B | 25-76 | 45.2 ± 8.5 | 5.8 | 52.4 | passive |
| 2019 | 2-4 | 1109 | B | 22-71 | 45.4 ± 8.1 | 1.3 | 48.2 | active |
| 2019 | 2-4 | 589 | B | 26-71 | 48.0 ± 9.4 | <1 | 58.7 | passive |
