# Supplementary material for: Living apart together: Long-term coexistence of Baltic cod stocks associated with depth-specific habitat use
Source: PLoS One. 2022 Sep 28;17(9):e0274476. doi: 10.1371/journal.pone.0274476 (PMC9518848; doi:10.1371/journal.pone.0274476)
Supplement: S3 Table — Definition of table headers and items as in S1 Table. (DOCX) [file pone.0274476.s009.docx]

| **Year/month(s)** | **N** | **Sampled areas** | **Length range [cm]** | **Mean length ± SD [cm]** | **Spawning fish [%]** | **Female fish [%]** | **WBC affiliation [%]** |
| --- | --- | --- | --- | --- | --- | --- | --- |
| 1979/11 | 26 | B,C | 31-69 | 47.1 ± 11.1 | 0 | 42.3 | 15.4 |
| 1983/12 | 23 | B,C | 33-61 | 48.1 ± 8.4 | 0 | 56.5 | 26.1 |
| 1985/1 | 47 | B | 31-72 | 50.9 ± 8.7 | 100 | 0 | 10.6 |
| 1986/1+2 | 12 | B,C | 46-69 | 54.3 ± 7.6 | 100 | 0 | 16.7 |
| 1986/12 | 15 | B,C | 30-52 | 41.6 ± 6.6 | 0 | 40.0 | 13.3 |
| 1987/1 | 3 | C | 43-50 | 47.0 ± 2.9 | 100 | 0 | 0 |
| 1988/2 | 8 | B,C | 34-53 | 44.8 ± 5.7 | 100 | 0 | 0 |
| 1989/1+2 | 30 | B,C | 35-64 | 47.8 ± 8.3 | 16.7 | 40.0 | 0 |
